# Anti-metastatic effect of methylprednisolone targeting vascular endothelial cells under surgical stress

**DOI:** 10.1038/s41598-021-85241-2

**Published:** 2021-03-18

**Authors:** Takaomi Hagi, Yukinori Kurokawa, Noboru Kobayashi, Tsuyoshi Takahashi, Takuro Saito, Kotaro Yamashita, Koji Tanaka, Tomoki Makino, Makoto Yamasaki, Kiyokazu Nakajima, Hidetoshi Eguchi, Yuichiro Doki

**Affiliations:** Department of Gastroenterological Surgery, Osaka University Graduate School of Medicine, 2-2, Yamadaoka, Suita, Osaka 565-0871 Japan

**Keywords:** Gastric cancer, Cancer microenvironment, Metastasis

## Abstract

Perioperative systemic inflammation induced by surgical stress elevates the risk of hematogenous cancer metastasis. This study investigated the anti-metastatic effects and mechanisms of methylprednisolone (MP) administration for surgical stress. We examined the effects of MP on the expression of adhesion molecules in human vascular endothelial cells and in a murine hepatic metastasis model under lipopolysaccharide (LPS) administration, which mimics systemic inflammation induced by surgical stress. Serum E-selectin level was measured in blood samples obtained from 32 gastric cancer patients who were randomly assigned to treat preoperatively with or without MP. The expression of E-selectin in LPS-induced vascular endothelial cells was suppressed by MP. An adhesion assay showed the number of LPS-induced adherent tumour cells was significantly lower following MP. In the in vivo study, LPS significantly elevated the number of hepatic metastases, but pretreatment with MP before LPS significantly inhibited this elevation. The LPS-induced expression of E-selectin in the vascular endothelium of the portal vein was suppressed by MP. In human clinical samples, serum E-selectin level was significantly decreased by preoperative MP. Suppression of surgically induced systemic inflammation by MP administration might prevent hematogenous cancer metastases by suppressing the induction of E-selectin expression in the vascular endothelium.

## Introduction

Surgical resection is a major treatment that can cure and control solid tumours. However, surgical stress itself accelerates tumour progression and the formation of distant metastases^1,2^. Several studies reported that the degree of surgical stress or severity of postoperative complications influenced the prognosis of cancer patients^3–5^. Recently, we have demonstrated that excessive surgical stress elevates the risk of hematogenous metastasis but not peritoneal or lymphatic metastasis in patients with gastric cancer^6^. A possible explanation for these results is that systemic inflammation caused by surgical stress promotes the expressions of cell adhesion molecules in the vascular endothelium of distant organs^7^. E-selectin, a major leukocyte adhesion receptor that is present on endothelial cells, plays detrimental roles in cell tethering and in the rolling interactions of tumour cells^8–10^. In addition, the expression of E-selectin induced by inflammatory stimuli facilitates the adhesion of circulating tumour cells, which increases hematogenous metastasis^11,12^. Despite these findings, only one study has focused on these adhesion molecules as a therapeutic target for tumour metastasis by suppressing inflammation induced by surgical stress^13^. We hypothesized that the suppression of surgically induced systemic inflammation might control the expression of E-selectin in the vascular endothelium, which might reduce postoperative cancer recurrence.

We previously reported that tumour growth promoted by administration of lipopolysaccharide (LPS), which causes systemic inflammation mimicking that induced by surgical stress, could be inhibited by pretreatment with methylprednisolone (MP), and safety of these drugs when administered to mice^14^. However, the effects of perioperative MP administration on tumour metastasis remain unclear. In this study, we used a murine model of colon cancer hepatic metastasis involving the intraperitoneal administration of LPS to determine whether MP had an effect on the number of hepatic metastases. In addition, we analyzed changes of expression in adhesion molecules, especially E-selectin, by administering MP both in vivo and in vitro following treatment with LPS. Moreover, serum E-selectin levels were examined using human clinical samples obtained from gastric cancer patients who were treated preoperatively with or without MP.

## Results

### Effect of MP on expression of adhesion molecules in vascular endothelial cells

**Figure 1:**
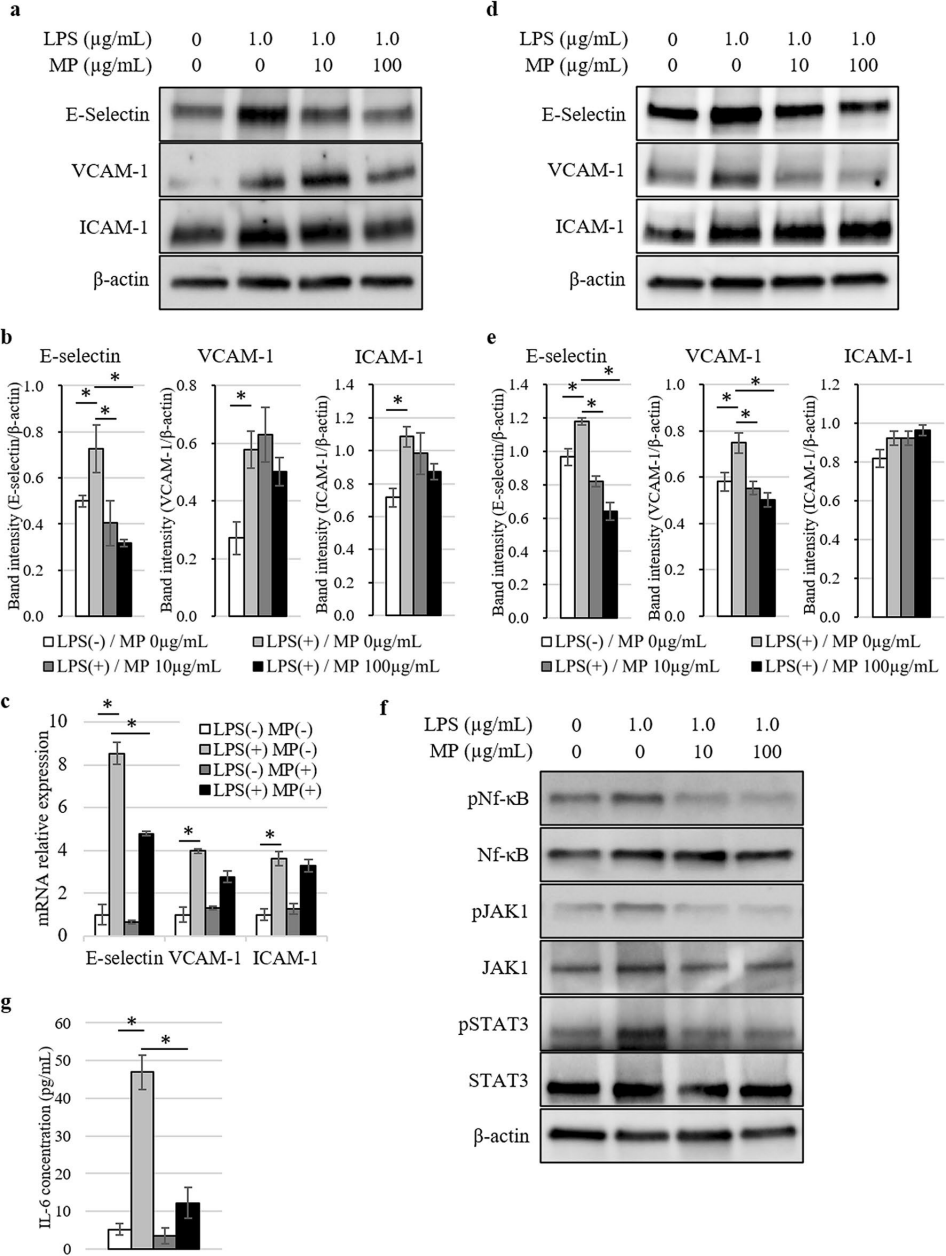
Analysis of adhesion molecules expressed in vascular endothelial cells stimulated by LPS and MP. (**a**) Protein expression levels of E-selectin, VCAM-1, and ICAM-1 in HUVEC at 6 h after stimulation analyzed by western blotting. Blot was cut horizontally and immunoblotting was performed for each section. (**b**) Quantification of E-selectin, VCAM-1, and ICAM-1 band intensity normalized to β-actin in HUVEC. Data are means ± standard error (n = 3, each group); *P < 0.05. (**c**) Induction of *SELE*, *VCAM1*, and *ICAM1* gene expression in HUVEC 6 h after stimulated by 1.0 µg/mL of LPS and 100 µg/mL of MP was analyzed by quantitative real-time PCR. Data are normalized relative to *ACTB* mRNA levels. Data are means ± standard error (n = 3, each group); *P < 0.05. (**d**) Protein expression levels of E-selectin, VCAM-1, and ICAM-1 in HHSEC at 6 h after stimulation analyzed by western blotting. Blot was cut horizontally and immunoblotting was performed for each section. (**e**) Quantification of E-selectin, VCAM-1, and ICAM-1 band intensity normalized to β-actin in HHSEC. Data are means ± standard error (n = 3, each group); *P < 0.05. (**f**) The phosphorylation of Nf-κB, JAK1, and STAT3 was analyzed by western blotting with antibodies targeting pNf-κB/Nf-κB, pJAK1/JAK1, and pSTAT3/STAT3 at 30 min after the stimulation. Blot was cut horizontally and immunoblotting was performed for each section. (**g**) ELISA analysis of IL-6 concentration levels in conditioned medium 6 h after stimulated by 1.0 µg/mL of LPS and 100 µg/mL of MP. Data are means ± standard error (n = 3, each group); *P < 0.05.

First, we investigated the effects of LPS and MP on the expression of major adhesion molecules in human umbilical vein endothelial cells (HUVEC) and human hepatic sinusoidal endothelial cells (HHSEC). At 6 h after stimulation with LPS, both western blotting and real-time PCR demonstrated increased expression of E-selectin, vascular cell adhesion molecule-1 (VCAM-1), and intercellular adhesion molecule-1 (ICAM-1) in HUVEC (Fig. 1a–c). The expression of E-selectin induced by LPS was strongly suppressed by MP administration, whereas suppressions of VCAM-1 and ICAM-1 were limited. In addition, MP administration strongly suppressed the expression of E-selectin and VCAM-1 induced by LPS in the analysis of western blotting in HHSEC (Fig. 1d,e). Next, we evaluated the constitutive activation statuses of Nf-κB, JAK1, and STAT3 on HUVEC. As shown in Fig. 1f and Supplementary Fig. S1 online, Nf-κB, JAK1, and STAT3 were all phosphorylated by LPS stimulation and suppressed by MP administration. Moreover, the IL-6 concentration in culture supernatants from HUVEC was elevated by LPS and significantly downregulated by MP administration (Fig. 1g). On the other hand, TNF-α and IL-1β concentrations were undetectable even after LPS stimulation.

### Effect of MP on LPS-induced tumour cell adhesion and invasion

To investigate whether LPS and MP affected the number of tumour cells that adhered to vascular endothelial cells, we conducted a tumour cell adhesion assay. First, we evaluated the expression of lysosomal-associated membrane protein 1 (LAMP1), lysosomal-associated membrane protein 2 (LAMP2), death receptor 3 (DR3), integrin β1, and integrin β2, those of which are ligands of E-selectin, VCAM-1, or ICAM-1, in five gastric cancer cell lines (MKN45, OCUM1, NUGC3, AGS, KATO III), as shown in Supplementary Fig. S2a online. AGS showed relatively low expression of all these ligands, while other cell lines showed high expression of some of the ligands. Because some of these cell lines were free-floating, we chose AGS and NUCG3 for the adhesion assay.

**Figure 2:**
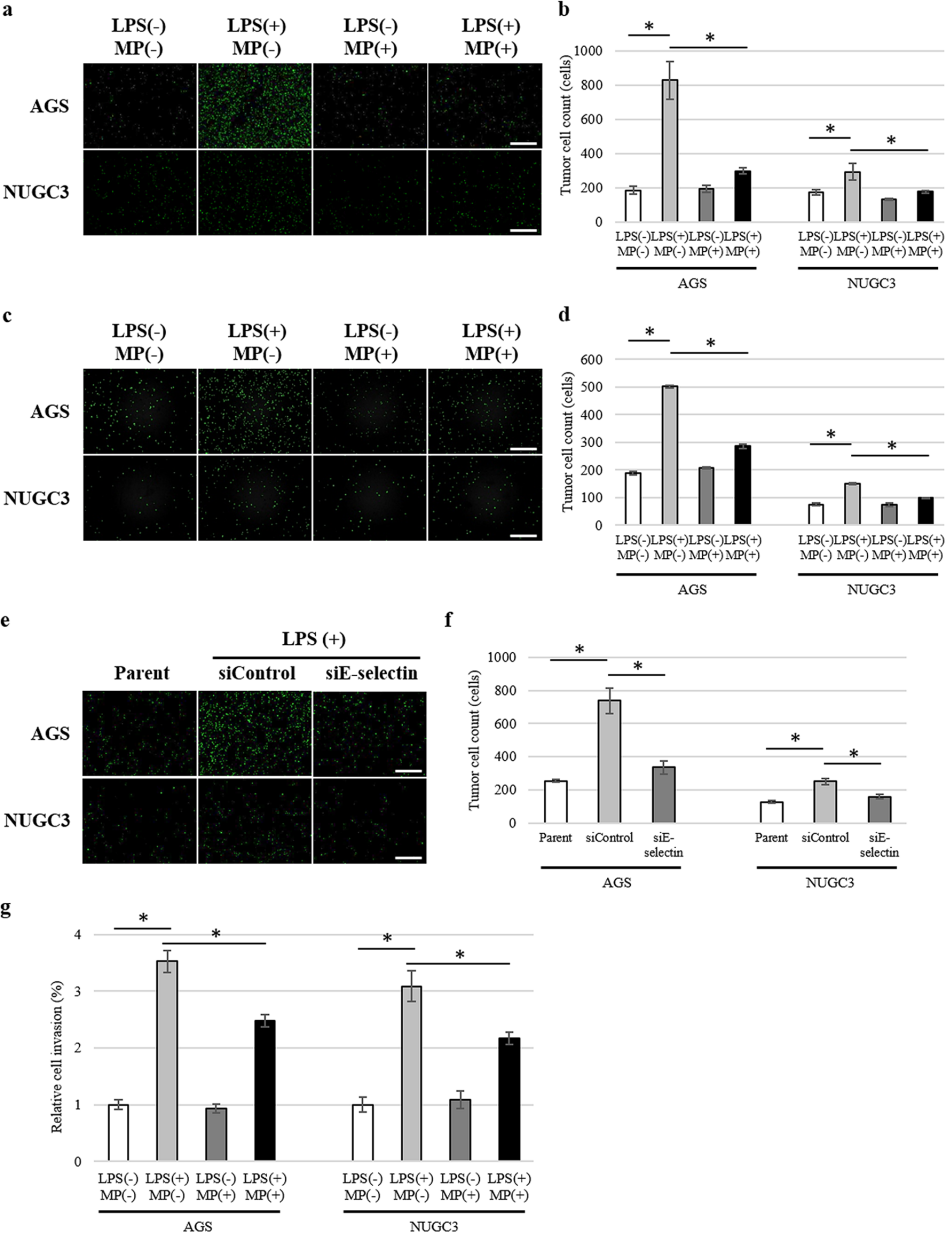
Adhesion of AGS and NUGC3 gastric cancer cells to vascular endothelial cells. (**a**) Representative images of the adhesion of tumour cells (AGS, upper; NUGC3, lower) to monolayer-cultured HUVEC stimulated by 1.0 µg/mL of LPS and 100 µg/mL of MP. Scale bars are equal to 500 µm. (**b**) Number of tumour-binding cells (AGS and NUGC3) to monolayer-cultured HUVEC stimulated by 1.0 µg/mL of LPS and 100 µg/mL of MP. Data are means ± standard error (n = 6, each group); *P < 0.05. (**c**) Representative images of the adhesion of tumour cells (AGS, upper; NUGC3, lower) to monolayer-cultured HHSEC stimulated by 1.0 µg/mL of LPS and 100 µg/mL of MP. Scale bars are equal to 500 µm. (**d**) Number of tumour-binding cells (AGS and NUGC3) to monolayer-cultured HHSEC stimulated by 1.0 µg/mL of LPS and 100 µg/mL of MP. Data are means ± standard error (n = 6, each group); *P < 0.05. (**e**) Representative images of the adhesion of tumour cells (AGS, upper; NUGC3, lower) to monolayer-cultured HUVEC depleted of E-selectin stimulated by 1.0 µg/mL of LPS. Scale bars are equal to 500 µm. (**f**) Number of tumour-binding cells (AGS and NUGC3) to monolayer-cultured HUVEC depleted of E-selectin stimulated by 1.0 µg/mL of LPS. Data are means ± standard error (n = 6, each group); *P < 0.05. (**g**) Invasion assay of of AGS and NUGC3 gastric cancer cells co-cultured with HUVEC stimulated by 1.0 µg/mL of LPS and 100 µg/mL of MP. Data are means ± standard error (n = 5, each group); *P < 0.05.

The number of tumour cells that adhered to LPS-stimulated HUVEC was significantly increased in both AGS and NUGC3 (Fig. 2a). Administration of MP significantly attenuated the increase in adherent tumour cells in both AGS (*P* = 0.001) and NUGC3 (*P* = 0.034; Fig. 2b). Similarly, administration of MP significantly attenuated the number of tumour cells that adhered to LPS-stimulated HHSEC in both AGS (*P* < 0.001) and NUGC3 (*P* < 0.001; Fig. 2c,d). To determine if expression of E-selectin played an important role in tumour cell adhesion and if LPS-induced tumour cell adhesion depended on E-selectin expression, we knocked down E-selectin in HUVEC and analyzed tumour cell adherence in both AGS and NUGC3 (Fig. 2e). The knockdown of E-selectin in HUVEC was confirmed by western blotting and real-time PCR (Supplementary Fig. S3 online). LPS-induced tumour cell adhesion to HUVEC was significantly inhibited by knockdown of E-selectin in both AGS (*P* = 0.001) and NUGC3 (*P* = 0.002; Fig. 2f).

To evaluate the transmigration of tumour cells through endothelial cells, we conducted an invasion assay by co-culturing HUVEC and tumour cells. Tumour invasion was significantly increased by LPS-stimulation (Fig. 2g). Administration of MP significantly attenuated the increase in tumour invasion in both AGS (*P* = 0.003) and NUGC3 (*P* = 0.025).

### Anti-metastatic effects of MP in hepatic metastasis models

**Figure 3:**
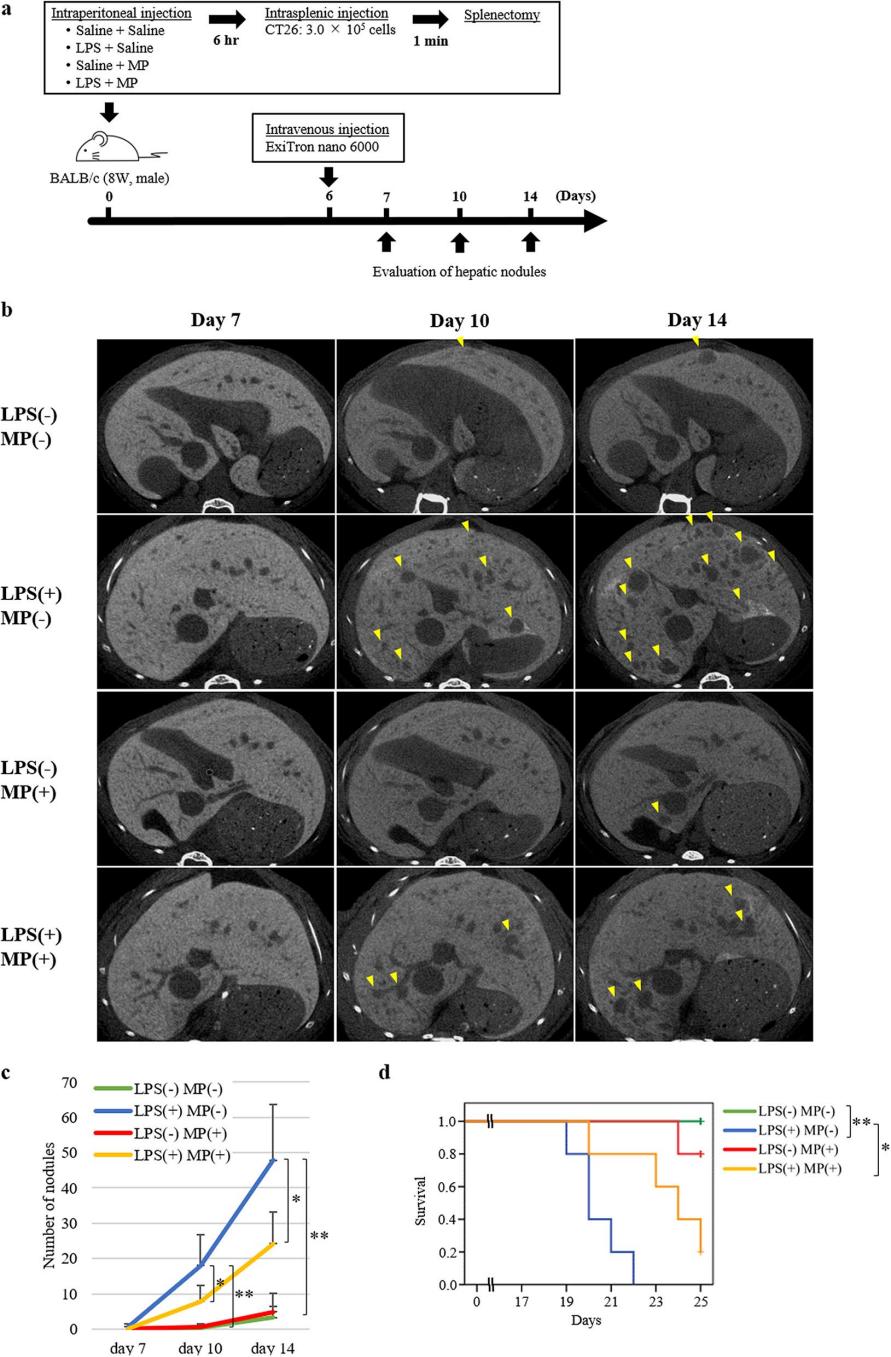
Suppressive effects of MP in LPS-induced murine models of hepatic metastasis. (**a**) Mouse colon cancer cell line (CT26) hepatic metastasis model; male BALB/c mice (8 weeks old) were treated with saline/saline (control), LPS/saline, saline/MP, or LPS/MP. Six hours later, 3.0 × 10^5^ CT26 cells were injected into the inferior pole of the spleen, followed by splenectomy 1 min later. The number of hepatic nodules was evaluated using enhanced micro-CT imaging on days 7, 10, and 14. (**b**) Representative CT images of the liver. Almost the same liver slice from the same individual in each group was photographed axially on days 7, 10, and 14. Yellow arrowheads indicate nodules with a diameter of more than 50 µm. (**c**) Metastatic nodules in the liver were quantified on days 7, 10, and 14. Data are means ± standard error (n = 5, each group); *P < 0.05, **P < 0.001. (**d**) Kaplan–Meier survival curves of each group of hepatic metastasis model mice. The horizontal axis represents the days from inoculation of tumour cells and the vertical axis represents the survival rate. Tick marks represent censored subjects. *Log-rank *P* < 0.05, **log-rank *P* < 0.01.

To confirm the anti-metastatic effect of MP on LPS-induced hepatic metastases, we examined the number of hepatic nodules over time. All mice were checked for health status, including microbiological status, before and during experiments and revealed no abnormalities. CT26 cells were used for this examination since CT26 treated with MP or LPS showed similar expression patterns with ligands of adhesion molecules to other human gastric cancer (Supplementary Fig. S2b online). Six hours after the intraperitoneal injection of MP or saline and LPS or saline, CT26 were inoculated intrasplenically. The number of hepatic metastases were evaluated on days 7, 10, and 14 using micro-CT imaging (Fig. 3a). No significant differences in the number of hepatic nodules were seen between the four groups on day 7 (Fig. 3b,c). In the LPS(+)/MP(−) group, the number of hepatic nodules was significantly higher than in the LPS(−)/MP(−) group on both day 10 (*P* = 0.001) and day 14 (*P* < 0.001). By contrast, administration of MP significantly reduced the number of LPS-induced hepatic nodules on both day 10 (*P* = 0.049) and day 14 (*P* = 0.020). The number of hepatic nodules was similar between the LPS(−)/MP(−) and LPS(−)/MP(+) groups throughout the observation period. Survival of hepatic metastasis model mice is shown in Fig. 3d. Survival was significantly worse in the LPS(+)/MP(−) group than in the LPS(−)/MP(−) group (log-rank *P* = 0.002), and was significantly better in the LPS(+)/MP(+) group than in the LPS(+)/MP(−) group (log-rank *P* = 0.016).

**Figure 4:**
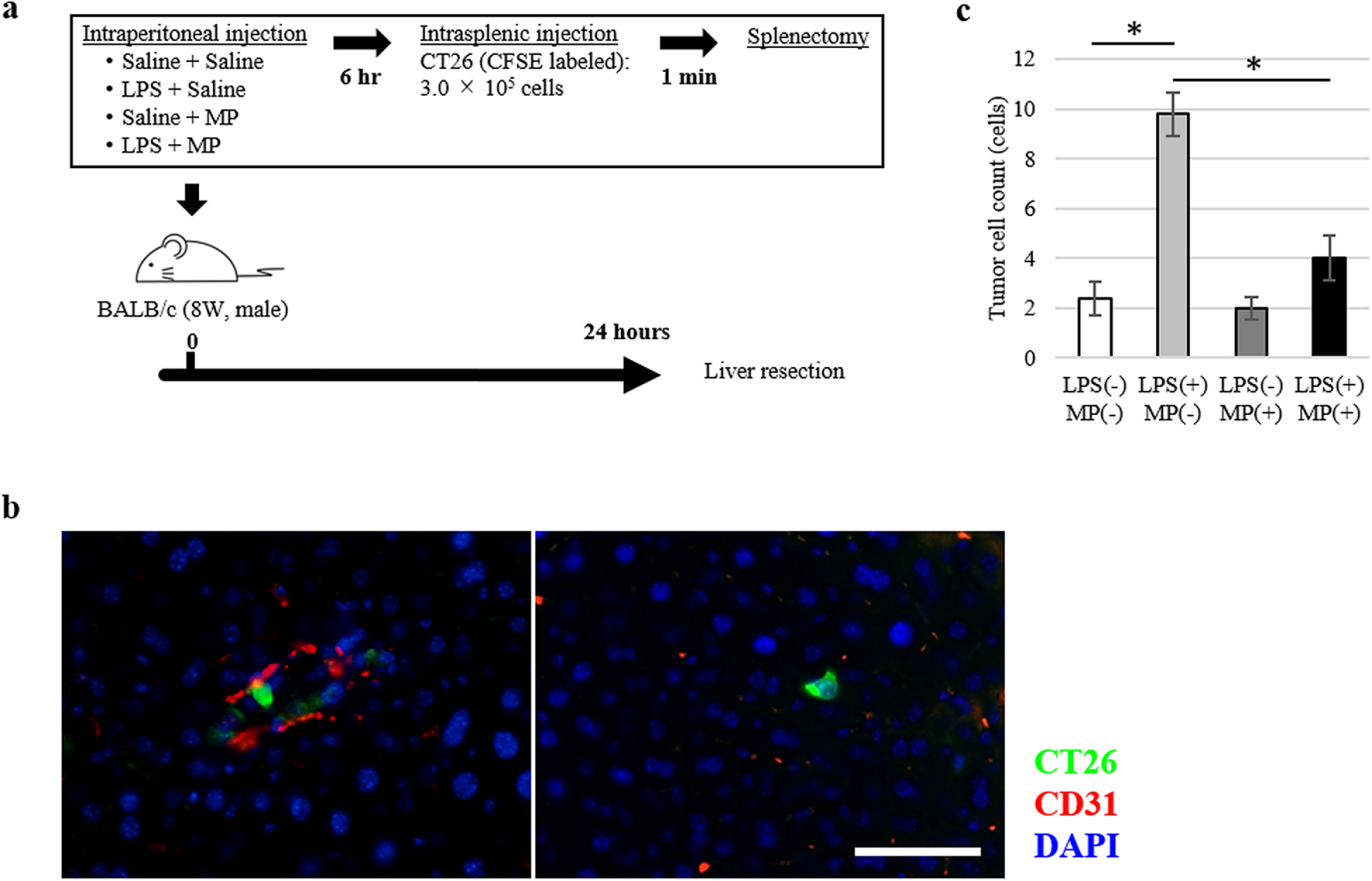
Suppressive effects of MP on tumour cell hepatic binding in LPS-induced murine hepatic metastasis models. (**a**) Mouse colon cancer cell line (CT26) hepatic metastasis model; male BALB/c mice (8 weeks old) were treated with saline/saline (control), LPS/saline, saline/MP, or LPS/MP. Six hours later, 3.0 × 10^5^ CT26 cells were injected into the inferior pole of the spleen, followed by splenectomy 1 min later. After 24 h, the liver was resected for analysis. (**b**) Representative images of CT26 tumour cells adhered to the endothelial cells (left) and transmigrated into the liver (right). CT26 tumour cells are labeled by CFSE fluorescence (green). Each protein was co-stained with anti-CD31 antibody to define vascular endothelial cells (red). Nuclei are stained with DAPI (blue). Scale bars are equal to 50 µm. (**c**) Number of CT26 tumour cells binding to the liver following pretreatment with LPS and MP. Data are means ± standard error (n = 5, each group); *P < 0.01.

To evaluate the early phase of tumour cell metastasis, we examined the number of tumour cells trapped to the liver 24 h after the inoculation of CT26 tumour cells (Fig. 4a). In the LPS(+)/MP(−) group, the number of tumour cells trapped to the liver was significantly higher than in the LPS(−)/MP(−) group (*P* < 0.001) (Fig. 4b,c, and Supplementary Fig. S4 online). By contrast, administration of MP significantly reduced the number of trapped tumour cells induced by LPS administration (*P* = 0.002).

### Effect of MP on hepatic microenvironment in vivo

**Figure 5:**
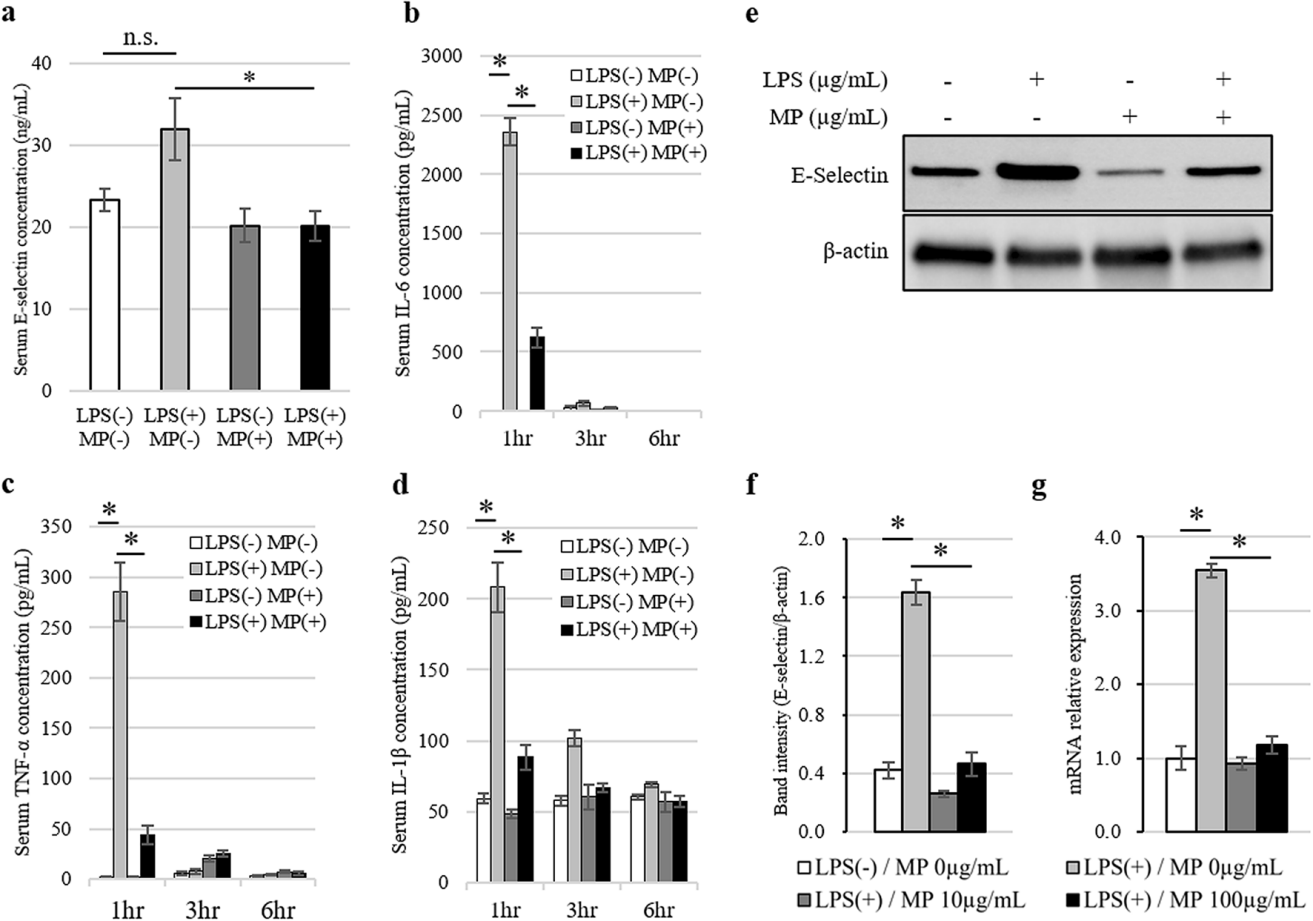
Analysis of E-selectin expressed in the serum and portal vein following MP administration in an LPS-induced murine hepatic metastasis model. (**a**) ELISA analysis of serum E-selectin levels at 6 h after stimulation. Data are means ± standard error (n = 4, each group); *P < 0.05. *n.s.* no significance. (**b**–**d**) Analysis of serum cytokine levels at 1, 3, and 6 h after stimulation with LPS and MP. ELISA analysis of serum concentrations of IL-6 (**b**), TNF-α (**c**), and IL-1β (**d**). Data are means ± standard error (n = 4, each group); *P < 0.01. (**e**) Western blotting analysis of E-selectin protein expression levels in the portal vein at 6 h after stimulation. Blot was cut horizontally and immunoblotting was performed for each section. (**f**) Quantification of E-selectin band intensity normalized to β-actin. Data are means ± standard error (n = 3, each group); *P < 0.05. (**g**) Induction of *Sele* gene expression at 6 h after stimulation was analyzed by quantitative real-time PCR. Data are normalized relative to *Actb* mRNA levels. Data are means ± standard error (n = 4, each group); *P < 0.05.

Next, we examined the effect of LPS and MP administration on E-selectin expressions in serum and local tissue samples in a murine model. Each sample was obtained and analyzed 6 h after the administration of LPS and MP. The levels of serum E-selectin were slightly elevated by LPS stimulation, although this change was not statistically significant (*P* = 0.071; Fig. 5a). On the other hand, MP administration significantly lowered the levels of serum E-selectin that had been elevated by LPS. Moreover, LPS stimulation significantly elevated the levels of serum cytokines (IL-6, TNF-α, and IL-1β), and this elevation was significantly attenuated by MP administration (Fig. 5b–d). In the portal vein, the expression of E-selectin was promoted by LPS stimulation and was suppressed by MP administration as shown by both western blotting and real-time PCR (Fig. 5e–g).

To examine the effect of LPS and MP administration on the recruitment of hematocytes, we performed immunofluorescence staining using the liver specimens (Supplementary Fig. S5a and S5b online). LPS stimulation significantly elevated the recruitment of neutrophils and platelets, and this elevation was significantly attenuated by MP administration (Supplementary Fig. S5c and S5d online).

### Effect of MP on postoperative serum E-selectin and IL-6 levels in human samples

**Figure 6:**
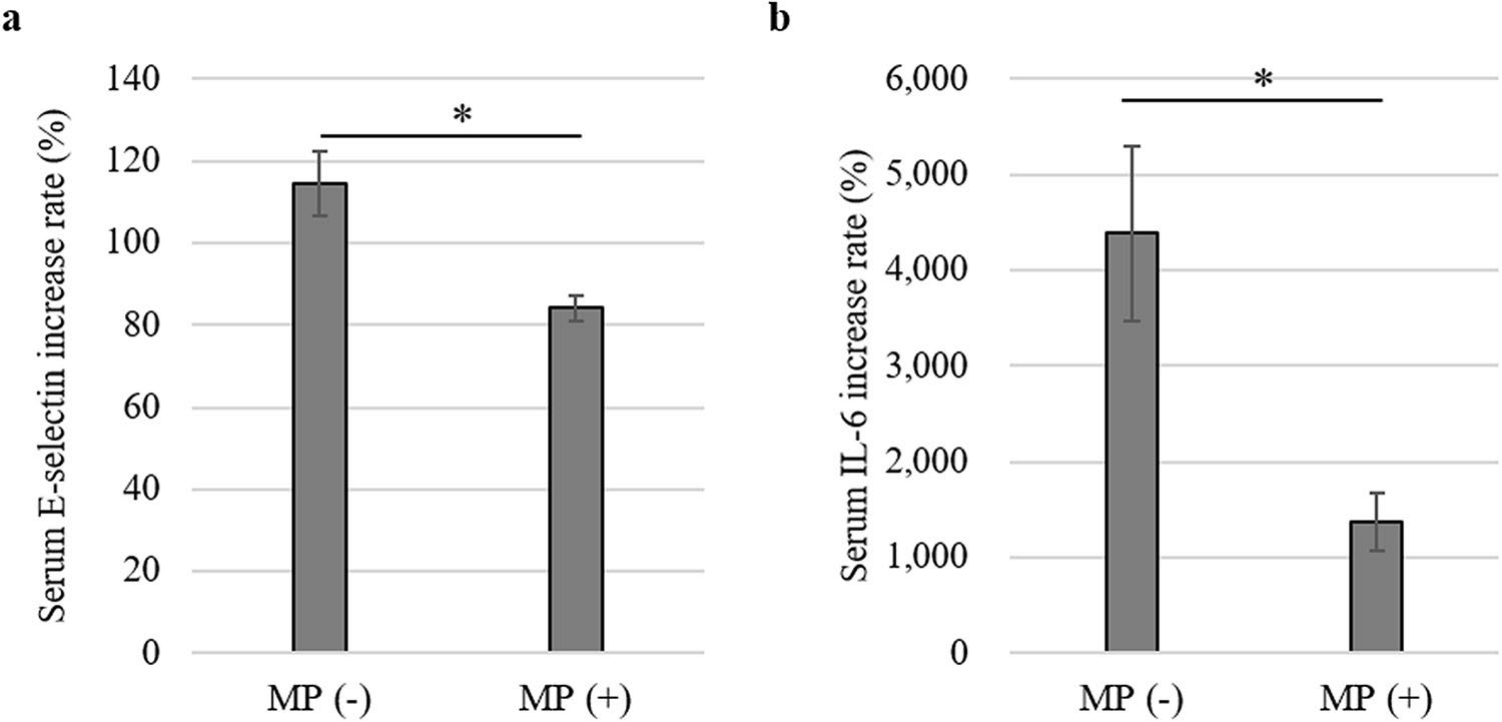
Effect of MP on postoperative serum molecular levels in human clinical samples. The percent increases from preoperative to postoperative samples of serum E-selectin (**a**) and IL-6 (**b**) levels are indicated. Data are means ± standard error (n = 17 for non-treated group and n = 15 for MP-treated group); *P < 0.01.

Of 33 patients who were enrolled in a randomized controlled trial from our institution, pre- and postoperative serum E-selectin and IL-6 levels samples were available from 32 patients. We examined these levels in 17 patients in the non-treated group and in 15 patients in the MP-treated group. The percent increases in E-selectin and IL-6 levels between before and after surgery were compared in each group. In the non-treated group, the mean increase in the serum E-selectin level was 114.5%, while that in the MP-treated group was 84.1%, indicating significant downregulation of serum E-selectin by preoperative MP administration (*P* = 0.002; Fig. 6a). In addition, the mean increase in the serum IL-6 level in the MP-treated group was significantly lower than that in the non-treated group (mean, 1379% vs. 4384%, respectively, *P* = 0.002; Fig. 6b).

## Discussion

Perioperative systemic inflammation is considered to be a predisposing factor for adhesion of tumour cells to vascular endothelial cells, which in turn increases the risk of hematogenous metastases^6^. However, there are few clinical treatments to prevent the acceleration of tumour metastasis due to surgical stress. In the in vivo portion of this study, we showed that the number of hepatic metastases induced by LPS administration was significantly reduced by the administration of MP without adverse events. In addition, the LPS-induced increase in the expressions of E-selectin in both the portal vein of the liver and in the serum was decreased by the administration of MP. In the in vitro portion of the study, we demonstrated that Nf-κB signaling and JAK/STAT signaling in HUVEC were possibly involved in the downregulation of LPS-induced E-selectin expression by MP. Moreover, we showed that MP inhibited LPS-induced tumour cell adhesion that was dependent on E-selectin expression. Furthermore, invasion of tumour cells induced by LPS was inhibited by the administration of MP. We also confirmed that preoperative administration of MP led to a significant decrease in serum E-selectin levels in human clinical samples. Taken together, this study suggests that MP administration during the perioperative period is a potential inhibitor of hematogenous tumour metastasis induced by surgical stress.

E-selectin is the major vascular cell adhesion receptor responsible for the initial rolling of circulating tumour cells and leukocytes and their attachment to the endothelium^15,16^. Several studies reported that the expression of E-selectin could be induced by inflammatory stimuli, including LPS and cytokines such as IL-1, IL-6, and TNF-α, peaking within 2–6 h after stimulation^17,18^. Moreover, E-selectin gene expression is regulated by both STAT3 and Nf-κB signaling^19,20^. Since surgical stress activates this signaling, we hypothesized that suppression of systemic inflammation induced by surgical stress could inhibit postoperative tumour metastasis. Systemic inflammation results from complicated effects of various cytokine and chemokine networks. Thus far, specific anti-cytokine antibodies or blockers have not adequately suppressed these reactions^21,22^. Accordingly, in this study we used MP, a strong anti-inflammatory drug that suppresses the activation of Nf-κB^23,24^. MP is the most commonly prescribed anti-inflammatory drug, and prophylactic administration of MP was shown to decrease postoperative morbidity and suppress the release of proinflammatory cytokines in surgery for esophageal cancer^25^. Thus, we examined whether MP could downregulate the expression of E-selectin in the context of a systemic inflammatory response and thereby reduce hepatic metastasis.

In this study, we proposed two possible mechanisms whereby MP administration might downregulate LPS-induced E-selectin expression. One is the suppressive effect of MP on Nf-κB signaling, which we demonstrated in the in vitro study. This suppression may directly inhibits the LPS-induced activation of Nf-κB signaling, which in turn induces E-selectin expression. Another mechanism might be the suppressive effect of MP on inflammatory cytokines induced by LPS. These cytokines could indirectly induce expression of E-selectin^26^. Indeed, our results demonstrated the suppressive effect of MP on IL-6 expression in both in vitro and in vivo experiments, as well as in human samples. We previously demonstrated this effect of MP on postoperative serum IL-6 levels in a smaller number of patients^14^. Although the precise mechanisms were not elucidated, these molecular are likely to play important roles.

The cascade of tumour metastasis is very intricate and requires a multistep mechanism of neoplastic progression, collectively termed the invasion–metastasis cascade^27^. For successful completion of this complicated process, it is important for a conducive microenvironment to form at a pre-metastatic site^28^. A previous study reported that NF-κB signaling established pre-metastatic sites in a manner dependent on Toll-like receptor 4, and that this inflammation-like state accelerated tumour metastases^29^. In addition, several studies revealed that inflammatory monocytes promoted formation of a pre-metastatic site by secreting VEGF^30,31^. Moreover, neutrophil extracellular traps, which are local snares formed by neutrophils that can trap circulating tumour cells, are promoted by surgical stress and are associated with the development of tumour recurrences in the liver^11,32,33^. Monocytes and platelets are also important regulators of neutrophil extracellular traps that associate with inflammatory stimulations^34,35^. All of these mechanisms are strongly associated with the inflammatory response, and may be suppressed by the administration of MP. In fact, we showed that the recruitment of neutrophils and platelets triggered by LPS was blocked by MP administration which might have effect on metastasis formation. We only focused mainly on the expression of E-selectin in the vascular endothelium, and conducted using a model without bearing a primary tumour, which was a major limitation of our study. Further studies are necessary to elucidate the detailed mechanism and role of MP administration in tumour metastasis.

There were several limitations to this study. The first is that we used LPS-induced inflammation as a model of systemic inflammation induced by surgical stress. While inflammation caused by surgical stress is mainly due to damage-associated molecular patterns, LPS results in typical pathogen-associated molecular patterns. However, these LPS-associated patterns commonly activate signal pathways such as NF-κB signaling, which is the main regulator of E-selectin expression. Nonetheless, several previous studies used LPS as a model of surgical stress^11–14^, and LPS administration increased both hepatic and lung metastases^11,13^. The second limitation is that we examined only two subtypes of gastric cancer cell lines in the in vitro experiment. Because the characteristics of tumour cells vary among subtypes, there might be subtypes for which MP administration during the perioperative period does not reduce the incidence of tumour metastases. The third limitation is that we examined only one tumor cell line (CT26) in the in vivo experiments. However, administration of LPS or MP to CT26 showed similar expression patterns of ligands of adhesion molecules to other human gastric cancer cell lines (AGS and NUGC3). Further studies should be conducted to identify tumour subtypes that will benefit from this treatment.

In conclusion, we demonstrated the anti-metastatic effect of MP administration in the context of systemic inflammation induced by surgical stress. Our findings indicated that MP suppressed E-selectin expression in the vascular endothelium, which inhibited tumour cell binding to endothelial cells. Since MP is the most commonly administered anti-inflammatory drug during the perioperative period, the application of these results may improve postoperative long-term outcomes.

## Methods

### Animal and cell lines

Eight-week-old male BALB/c mice (weight of 20–24 g) purchased from CLEA Japan (Tokyo, Japan) were used in this study. Animals were maintained under a temperature-controlled room (20–24 °C); 12 h dark/12 h light cycle; three adults per cage; gender is not mixed; free access to water and diets; high adsorbing power bedding materials which were changed every week; and specific pathogen free conditions in the laboratory of the Institute of Experimental Animal Sciences Faculty of Medicine, Osaka University. This study was carried out in compliance with the ARRIVE guidelines (https://arriveguidelines.org) and got the ethical approval from the Animal Experiment Committee of Osaka University (no. 30-028-004). All animal experiments were conducted according to the Animal (Scientific Procedures) Act 1986, and the institutional ethical guidelines for animal experimentation of Osaka University.

Human gastric cancer cell lines MKN45 (JCRB0254), OCUM-1 (JCRB0192), and NUGC3 (JCRB0822) were obtained from the Japanese Collection of Research Bioresources (Osaka, Japan), and AGS (CRL1739) and KATO III (HTB103) from the American Type Culture Collection (Rockville, MD, USA). Mouse colon adenocarcinoma cells (CT26.WT; ADCC CRL-2638) were obtained from the American Type Culture Collection. These cells were cultured in RPMI-1640 medium (Life Technologies, Carlsbad, CA, USA) supplemented with 10% fetal bovine serum (FBS, Sigma-Aldrich, St. Louis, MO, USA) and 1% penicillin and streptomycin (Life Technologies). HUVEC were obtained from Lonza (Walkersville, MD, USA) and cultured in EGM-2 (Lonza). HHSEC were obtained from ScienCell Research Laboratories (Carlsbad, CA, USA) and cultured in Endothelial Cell Medium (ScienCell Research Laboratories). All cells were incubated at 37 °C with 5% CO_2_.

For small interfering RNA (siRNA) knockdown, the cells were transfected with control siRNA (sc-37007) or E-selectin siRNA (sc-29296) from Santa Cruz Biotechnology (Dallas, TX, USA) using Lipofectamine RNAiMAX transfection reagent (Life Technologies).

### Western blot analysis

All cell lines were starved for 24 h. AGS, NUGC3, HUVEC, and HHSEC were treated with 10 or 100 µg/mL of MP and 1.0 µg/mL of LPS for either 30 min or 6 h. Cells were lysed in lysis buffer on ice and collected by scraping. For preparation of mouse portal vein lysate, portal veins were dissected and homogenized in lysis buffer. After insoluble debris was removed by centrifugation, protein concentrations were quantified by the Bradford protein assay (Bio-Rad Laboratories, Hercules, CA, USA). For each sample, 10 µg of each protein was separated by 12% SDS-PAGE gels (Bio-Rad Laboratories) and transferred to PVDF membranes. The primary and secondary antibodies used are summarized in Supplementary Table S1 online. After incubation with secondary antibodies and ECL Western Blotting Detection Reagent (GE Healthcare Biosciences, Little Chalfont, UK), chemiluminescence was detected by ChemiDoc Touch Imaging System (Bio-Rad Laboratories). The experiment was performed at least in triplicate and the bands were quantified by densitometry using Image Lab software Version 6.0.0 (Bio-Rad Laboratories).

### RNA extraction and quantitative real-time PCR

Total RNA was extracted from mouse portal veins or cell lines using TRIzol RNA Isolation Reagent (Thermo Fisher Scientific, Waltham, MA, USA). cDNAs were synthesized using a Reverse Transcription System (Promega, Madison, WI, USA). The sequences of the PCR primers are shown in Supplementary Table S2 online. The quantitative real-time PCR analyses were performed with Thunderbird SYBR qPCR Mix (TOYOBO). PCR products were measured continuously using the 7900HT Fast Real-Time PCR System (Applied Biosystems, Hercules, CA, USA). The values were normalized to β-actin expression levels.

### Enzyme-linked immunosorbent assay

Protein levels of mouse serum E-selectin, interleukin-6 (IL-6), and tumour necrosis factor-α (TNF-α) were measured using enzyme-linked immunosorbent assay (ELISA) kits from R&D Systems, and IL-1β was assessed with an ELISA kit from RayBiotech (Norcross, GA, USA). The cell culture supernatants were harvested and centrifuged at 14,000*g* to remove the cellular debris. Protein levels of IL-6, TNF-α, and IL-1β were measured using a Human Quantikine ELISA kit (R&D Systems).

### Tumour cell adhesion assay

To quantify tumour cell adhesion (AGS or NUGC3) to HUVEC and HHSEC, we used a modified version of a previously described cell adhesion assay^13^. Briefly, a confluent monolayer of cultured HUVEC and HHSEC were treated with PBS/PBS, LPS (1.0 µg/mL)/PBS, PBS/MP (100 µg/mL), or LPS/MP for 30 min and then washed twice with EGM-2 medium containing 1% FBS. After 6 h of incubation, AGS or NUGC3 tumour cells (5.0 × 10^4^ cells per dish), labeled using a CFSE fluorescent kit (ab113853, Abcam), were added and co-cultured for 30 min. The dishes were then washed three times with PBS and fixed with 4% paraformaldehyde. The number of fluorescent-labeled tumour cells was counted from six independent sections using a BZ-X710 fluorescence microscope and its Hybrid Cell Count software (KEYENCE, Itasca, IL, USA). In addition, the number of tumour cells adhering to E-selectin depleted HUVEC with or without LPS stimulation was also analyzed.

### Invasion assay

An invasion assay was performed using Corning BioCoat Matrigel invasion chambers (Becton–Dickinson Labware, Bedford, MA, USA). Briefly, HUVEC was seeded 1.0 × 10^4^ cells per well in 24-well plates and cultured in EGM-2 for 30 min. After the incubation, AGS or NUGC3 tumour cells (3.0 × 10^4^ cells per well) treated with PBS/PBS, LPS (1.0 µg/mL)/PBS, PBS/MP (100 µg/mL), or LPS/MP were added and incubated at 37 °C for 24 h. The non-invading cells on the upper surface on the membrane were then detached. The invading cells were fixed and stained using a Diff-QuikTM Kit (BD Biosciences, Bedford, MA, USA). The number of tumour cells was counted from five independent sections using a microscope.

### Experimental hepatic metastasis models

Mice were anesthetized using isoflurane in each model. Intraperitoneal administration of LPS mimics systemic inflammation induced by surgical stress, as previously described^11–14^. A total of 20 mice were classified randomly into four groups: saline/saline (n = 5), LPS/saline (n = 5), saline/MP (n = 5), and LPS/MP (n = 5), using the sequentially numbered container method. Mice were first injected intraperitoneally with 100 µL of MP (10 mg/kg) or saline, then with 100 µL of LPS (10 µg/kg) or saline. Six hours later, tumour cells (CT26) were inoculated. Spleens were exposed through a 5-mm incision in the left hypochondrium, then 3.0 × 10^5^ CT26 cells in 100 µL PBS were inoculated into the inferior pole of the spleen and the mice were splenectomized 1 min later. One hundred microliters of ExiTron nano 6000 (Miltenyi Biotec, Bergisch-Gladbach, Germany), a contrast agent for small animals, was injected into the tail vein 6 days after the tumour inoculation. To evaluate the number of hepatic metastases, mice were anesthetized and photographed using micro-CT imaging (R_mCT2; Rigaku Corporation, Tokyo, Japan) at settings of 90 kV and 160 μA on days 7, 10, and 14. The SimpleViewer software program (Rigaku) was used for the image analysis. Nodules with an axial diameter of more than 50 µm were counted while blind to the assigned groups. Survival of these mice was examined until 25 days after the inoculation of tumour cells. No criteria were set for exclusion of mice and all the mice were included in the analysis. Mice were housed in a home cage during the experiment and monitored every day.

To analyze the early phase of tumour cells trapped to the liver, CT26 cells were labeled with a CFSE fluorescent kit before inoculation into the spleen, as described above. The following day, after killing by standard CO_2_ asphyxiation, the liver was resected and fixed with 4% paraformaldehyde. Liver specimens were sectioned to 4-µm thickness and immunofluorescence staining was performed as previously described^36^. The primary and secondary antibodies used are summarized in Supplementary Table S3 online. The nuclei were stained with DAPI (P36983; Thermo Fisher Scientific), and the number of fluorescent-labeled tumour cells was counted from five independent sections using the BZ-X710 fluorescence microscope and its Hybrid Cell Count software.

For the analysis of molecular expressions at the time of tumour cell inoculation, samples were obtained from the blood, liver, and portal vein.

### Human clinical samples

We obtained blood samples from patients with resectable gastric cancer who enrolled in a randomized controlled trial evaluating the efficacy of preoperative MP administration (UMIN000024465, 18/10/2016). Patients were randomly assigned to either the non-treated group or the MP-treated group which received preoperative intravenous administration of 5 mg/kg of MP once immediately before the skin incision. We examined serum E-selectin and IL-6 levels, both preoperatively (within 2 weeks before surgery) and postoperatively (the day after surgery) only in patients enrolled at our institution between December 2016 and October 2018. Levels of serum E-selectin and IL-6 were measured by a clinical laboratory testing service (SRL, Tokyo, Japan). Written informed consent was obtained from all patients. This study was performed in accordance with the Declaration of Helsinki and approved by the Institutional Review Board of Osaka University Hospital (nos. 18333, 19243).

### Statistical analysis

Data are expressed as the mean ± standard error. Statistical differences were evaluated using Student’s *t*-test. Two-sided *P* values were calculated and a value of *P* < 0.05 was considered statistically significant. Survival of hepatic metastasis model mice was defined as the interval from the date of tumour cell inoculation to the date of death. Survival rates were estimated using the Kaplan–Meier method and were compared with the log-rank test. All statistical analyses were performed with SPSS software, version 22.0 (IBM Corp., Armonk, NY, USA).

## Supplementary Information

Supplementary Information.

## Data Availability

All data and materials are available in this study.

## References

[1] Tohme S, Simmons RL, Tsung A (2017). Surgery for cancer: A trigger for metastases. Cancer Res..

[2] van der Bij GJ (2009). The perioperative period is an underutilized window of therapeutic opportunity in patients with colorectal cancer. Ann. Surg..

[3] Artinyan A (2015). Infectious postoperative complications decrease long-term survival in patients undergoing curative surgery for colorectal cancer: A study of 12,075 patients. Ann. Surg..

[4] Lerut T (2009). Postoperative complications after transthoracic esophagectomy for cancer of the esophagus and gastroesophageal junction are correlated with early cancer recurrence: Role of systematic grading of complications using the modified Clavien classification. Ann. Surg..

[5] Saito T (2015). Which is a more reliable indicator of survival after gastric cancer surgery: Postoperative complication occurrence or C-reactive protein elevation?. J. Surg. Oncol..

[6] Kurokawa Y (2020). Prognostic value of postoperative C-reactive protein elevation versus complication occurrence: A multicenter validation study. Gastric Cancer.

[7] De Martin R, Hoeth M, Hofer-Warbinek R, Schmid JA (2000). The transcription factor NF-κB and the regulation of vascular cell function. Arterioscler. Thromb. Vasc. Biol..

[8] Ley K (2003). The role of selectins in inflammation and disease. Trends Mol. Med..

[9] Ludwig RJ, Schon MP, Boehncke WH (2007). P-selectin: A common therapeutic target for cardiovascular disorders, inflammation and tumour metastasis. Expert Opin. Ther. Targets.

[10] Witz IP (2008). The selectin-selectin ligand axis in tumor progression. Cancer Metastasis Rev..

[11] McDonald B (2009). Systemic inflammation increases cancer cell adhesion to hepatic sinusoids by neutrophil mediated mechanisms. Int. J. Cancer.

[12] Jiang M (2014). Systemic inflammation promotes lung metastasis via E-selectin upregulation in mouse breast cancer model. Cancer Biol. Ther..

[13] Nojiri T (2015). Atrial natriuretic peptide prevents cancer metastasis through vascular endothelial cells. Proc. Natl. Acad. Sci. U.S.A..

[14] Taniguchi Y (2019). Methylprednisolone inhibits tumor growth and peritoneal seeding induced by surgical stress and postoperative complications. Ann. Surg. Oncol..

[15] Brodt P (1997). Liver endothelial E-selectin mediates carcinoma cell adhesion and promotes liver metastasis. Int. J. Cancer.

[16] Sperandio M, Gleissner CA, Ley K (2009). Glycosylation in immune cell trafficking. Immunol. Rev..

[17] Bevilacqua MP, Pober JS, Mendrick DL, Cotran RS, Gimbrone MA (1987). Identification of an inducible endothelial-leukocyte adhesion molecule. Proc. Natl. Acad. Sci. U.S.A..

[18] Haraldsen G, Kvale D, Lien B, Farstad IN, Brandtzaeg P (1996). Cytokine-regulated expression of E-selectin, intercellular adhesion molecule-1 (ICAM-1), and vascular cell adhesion molecule-1 (VCAM-1) in human microvascular endothelial cells. J. Immunol..

[19] Kim KJ (2017). STAT3 activation in endothelial cells is important for tumor metastasis via increased cell adhesion molecule expression. Oncogene.

[20] Ishii H, Takada K (2002). Bleomycin induces E-selectin expression in cultured umbilical vein endothelial cells by increasing its mRNA levels through activation of NF-kappaB/Rel. Toxicol. Appl. Pharmacol..

[21] Steven M (1997). Confirmatory interleukin-1 receptor antagonist trial in severe sepsis: A phase III, randomized, double-blind, placebo-controlled, multicenter trial. Crit. Care Med..

[22] Cohen J, Carlet J (1996). INTERSEPT: An international, multicenter, placebo-controlled trial of monoclonal antibody to human tumor necrosis factor-alpha in patients with sepsis. Crit. Care Med..

[23] Ray A, Prefontaine KE (1994). Physical association and functional antagonism between the p65 subunit of transcription factor Nf-κB and the glucocorticoid receptor. Proc. Natl. Acad. Sci. U.S.A..

[24] Brostjan C, Anrather J, Csizmadia V, Natarajan G, Winkler H (1997). Glucocorticoids inhibit E-selectin expression by targeting NF-kappaB and not ATF/c-Jun. J. Immunol..

[25] Sato N (2002). Randomized study of the benefits of preoperative corticosteroid administration on the postoperative morbidity and cytokine response in patients undergoing surgery for esophageal cancer. Ann. Surg..

[26] Barthel SR, Gavino JD, Descheny L, Dimitroff CJ (2007). Targeting selectins and selectin ligands in inflammation and cancer. Expert Opin. Ther. Targets.

[27] Valastyan S, Weinberg RA (2011). Tumor metastasis: Molecular insights and evolving paradigms. Cell.

[28] Psaila B, Lyden D (2009). The metastatic niche: Adapting the foreign soil. Nat. Rev. Cancer.

[29] Hiratsuka S (2008). The S100A8-serum amyloid A3-TLR4 paracrine cascade establishes a premetastatic phase. Nat. Cell Biol..

[30] Qian BZ (2011). CCL2 recruits inflammatory monocytes to facilitate breast-tumour metastasis. Nature.

[31] Hara T (2017). Control of metastatic niche formation by targeting APBA3/Mint3 in inflammatory monocytes. Proc. Natl. Acad. Sci. U.S.A..

[32] Tohme S (2016). Neutrophil extracellular traps promote the development and progression of liver metastases after surgical stress. Cancer Res..

[33] Cools-Lartigue J (2013). Neutrophil extracellular traps sequester circulating tumor cells and promote metastasis. J. Clin. Investig..

[34] von Brühl ML (2012). Monocytes, neutrophils, and platelets cooperate to initiate and propagate venous thrombosis in mice in vivo. J. Exp. Med..

[35] Clark SR (2007). Platelet TLR4 activates neutrophil extracellular traps to ensnare bacteria in septic blood. Nat. Med..

[36] Hwang JA (2009). COMP-Ang1 potentiates the antitumor activity of 5-fluorouracil by improving tissue perfusion in murine Lewis lung carcinoma. Mol. Cancer Res..

