# Supplementary material for: Anti-metastatic effect of methylprednisolone targeting vascular endothelial cells under surgical stress
Source: Sci Rep. 2021 Mar 18;11:6268. doi: 10.1038/s41598-021-85241-2 (PMC7973421; doi:10.1038/s41598-021-85241-2)
Supplement: Supplementary file 1 — Supplementary Information. [file 41598_2021_85241_MOESM1_ESM.docx]

**Anti-metastatic effect of methylprednisolone targeting vascular endothelial cells under surgical stress**

Takaomi Hagi^1^, Yukinori Kurokawa^1*^, Noboru Kobayashi^1^, Tsuyoshi Takahashi^1^, Takuro Saito^1^, Kotaro Yamashita^1^, Koji Tanaka^1^, Tomoki Makino^1^, Makoto Yamasaki^1^, Kiyokazu Nakajima^1^, Hidetoshi Eguchi^1^, Yuichiro Doki^1^

^1^ Department of Gastroenterological Surgery, Osaka University Graduate School of Medicine, Osaka, Japan.

* Corresponding author: Yukinori Kurokawa, MD, PhD

**Supplementary Table S1.** Antibodies used in Western blot analysis

**Supplementary Table S2.** Quantitative RT-PCR primers

**Supplementary Table S3.** Antibodies used in immunofluorescence staining

**Supplementary Fig. S1.** Quantification of (a) phospho-Nf-κB, (b) phospho-JAK1, and (c) phospho-STAT3 band intensity normalized to Nf-κB, JAK1, and STAT3, respectively, in HUVEC. Data are means ± standard error (n = 3, each group); **P* < 0.05.

**Supplementary Fig. S2.** Protein expression levels of ligands of E-selectin, vascular cell adhesion molecule-1, and intercellular adhesion molecule-1, specifically LAMP1, LAMP2, DR3, integrin β1, and integrin β2, were analyzed by western blotting. Blot was cut horizontally and immunoblotting was performed for each section. (a) Analysis of each ligand expressed in gastric cancer cell lines (MKN45, OCUM-1, NUGC3, AGS, and KATO III). (b) Analysis of changes in the expression of each ligand in AGS, NUGC3, and CT26 following stimulation by LPS and MP.

**Supplementary Fig. S3.** Confirmation of diminished E-selectin expression in HUVEC. (a) Protein expression levels of E-selectin were analyzed by western blotting. Blot was cut horizontally and immunoblotting was performed for each section. (b) Induction of *SELE* gene expression was analyzed by quantitative real-time PCR. Data are normalized relative to *ACTB* mRNA levels. Data are means ± standard error (n = 5, each group); **P* < 0.05.

**Supplementary Fig. S4.** Representative images of CT26 tumour cells trapped to the liver. CT26 tumour cells are labeled by CFSE fluorescence (green). White arrowheads indicate tumour cells labeled by CFSE fluorescence. Nuclei are stained with DAPI (blue). Scale bars are equal to 200 µm. Each image is representative of three independent experiments.

**Supplementary Fig. S5.** Representative images of (a) Ly6G+ neutrophil (green) and (b) CD42b+ platelet (green) recruitment in the liver. Each protein was co-stained with CD31 to define vascular endothelial cells (red). Nuclei were stained with DAPI (blue). Scale bars are equal to 50 µm. Number of (c) Ly6G and (d) CD42b positive cells following pretreatment with LPS and MP. Data are means ± standard error (n = 5, each group); *P < 0.05.

**Supplementary Fig. S6.** Uncropped blots indicated in (a) Figure 1a, (b) Figure 1d, (c) Figure 1f, (d) Figure 5e, (e) Supplementary Figure S2a, (f) Supplementary Figure S2b, and (g) Supplementary Figure S3.

**Supplementary Table S1.**

**Antibodies used in Western blot analysis**

| Antigen | Manufacturer | Catalog no. | Dilution |
| --- | --- | --- | --- |
| Primary antibody |  |  |  |
| phospho-Nf-κB | Cell Signaling Technology | #3033 | 1:500 |
| Nf-κB | Cell Signaling Technology | #8242 | 1:1,000 |
| phospho-JAK1 | Cell Signaling Technology | #3331 | 1:500 |
| JAK1 | Cell Signaling Technology | #3332 | 1:1,000 |
| phospho-STAT3 | Cell Signaling Technology | #9145 | 1:500 |
| STAT3 | Cell Signaling Technology | #9139 | 1:1,000 |
| E-selectin | Santa Cruz Biotechnology | sc137054 | 1:500 |
| VCAM-1 | Santa Cruz Biotechnology | sc18864 | 1:500 |
| ICAM-1 | Santa Cruz Biotechnology | sc390483 | 1:500 |
| Integrin β1 | Santa Cruz Biotechnology | sc374429 | 1:500 |
| Integrin β2 | Santa Cruz Biotechnology | sc-8420 | 1:500 |
| LAMP1 | Abcam | ab24170 | 1:500 |
| LAMP2 | Abcam | ab25631 ab13524 | 1:500  1:500 |
| DR3/TNFRSF25 | R&D Systems | #59204 | 1:500 |
| β-actin | Sigma-Aldrich | A2066 | 1:1,000 |
| Secondary antibody |  |  |  |
| Rabbit IgG HRP Linked  Whole Ab | GE Healthcare | NA934V | 1:10,000-100,000 |
| Mouse IgG HRP Linked  Whole Ab | GE Healthcare | NA931V | 1:10,000-100,000 |
| Anti-rat IgG, HRP-linked Antibody | Cell Signaling Technology | #7077 | 1: 100,000 |

**Supplementary Table S2.**

**Quantitative RT-PCR primers**

| **Gene** | **Species** | **Forward Primers (5’ to 3’)** | **Reverse Primers (5’ to 3’)** |
| --- | --- | --- | --- |
| *SELE* | Human | CTCGGACATGTGGAGCCACAGGACA | GGCTTTGGCAGCTGCTGGCAGGAAC |
| *VCAM1* | Human | GGAAAAGTTCTTGTTTGCCGAGCTA | GGAGCTGGTAGACCCTCGCTGGAAC |
| *ICAM1* | Human | GGCAAGAACCTTACCCTACGCTGCC | GTTCAGTGCGGCACGAGAAATTGGC |
| *ACTB* | Human | AGAGCTACGAGCTGCCTGAC | AGCACTGTGTTGGCGTACAG |
| *Sele* | Mouse | GAGCTCAGAATCTACAGTGTACCTC | GGATTTGTGGTGTCCACTGCCCTTG |
| *Actb* | Mouse | CATTGCTGACAGGATGCAGAAGG | TGCTGGAAGGTGGACAGTGAGG |

**Supplementary Table S3.**

**Antibodies used in immunofluorescence staining**

| Antigen | Manufacturer | Catalog no. | Dilution |
| --- | --- | --- | --- |
| Primary antibody |  |  |  |
| Mouse Ly6G (Gr-1) antibody | R&D Systems | MAB10371 | 1:100 |
| Anti-CD42b antibody [SP219] | Abcam | ab183345 | 1:200 |
| Anti-CD31 antibody | Abcam | ab28364 | 1:50 |
| Secondary antibody |  |  |  |
| Goat Anti-Mouse IgG H&L  (Alexa Fluor® 647) | Abcam | Ab150115 | 1:800 |
| Goat Anti-Rabbit IgG H&L  (Alexa Fluor® 750) | Abcam | ab175734 | 1:800 |

**
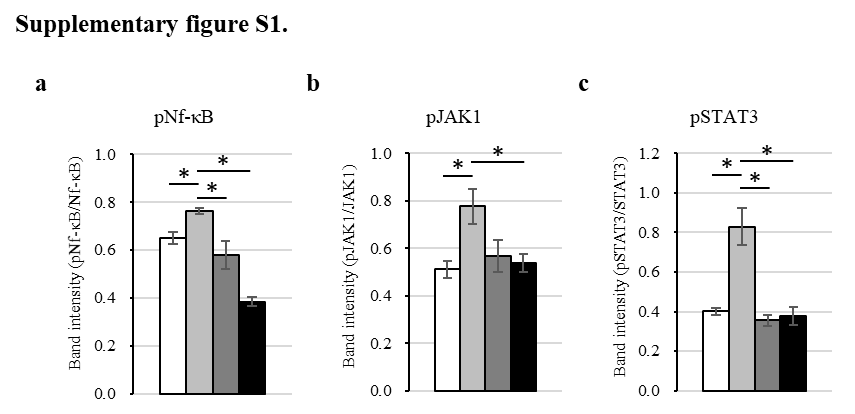
**

**
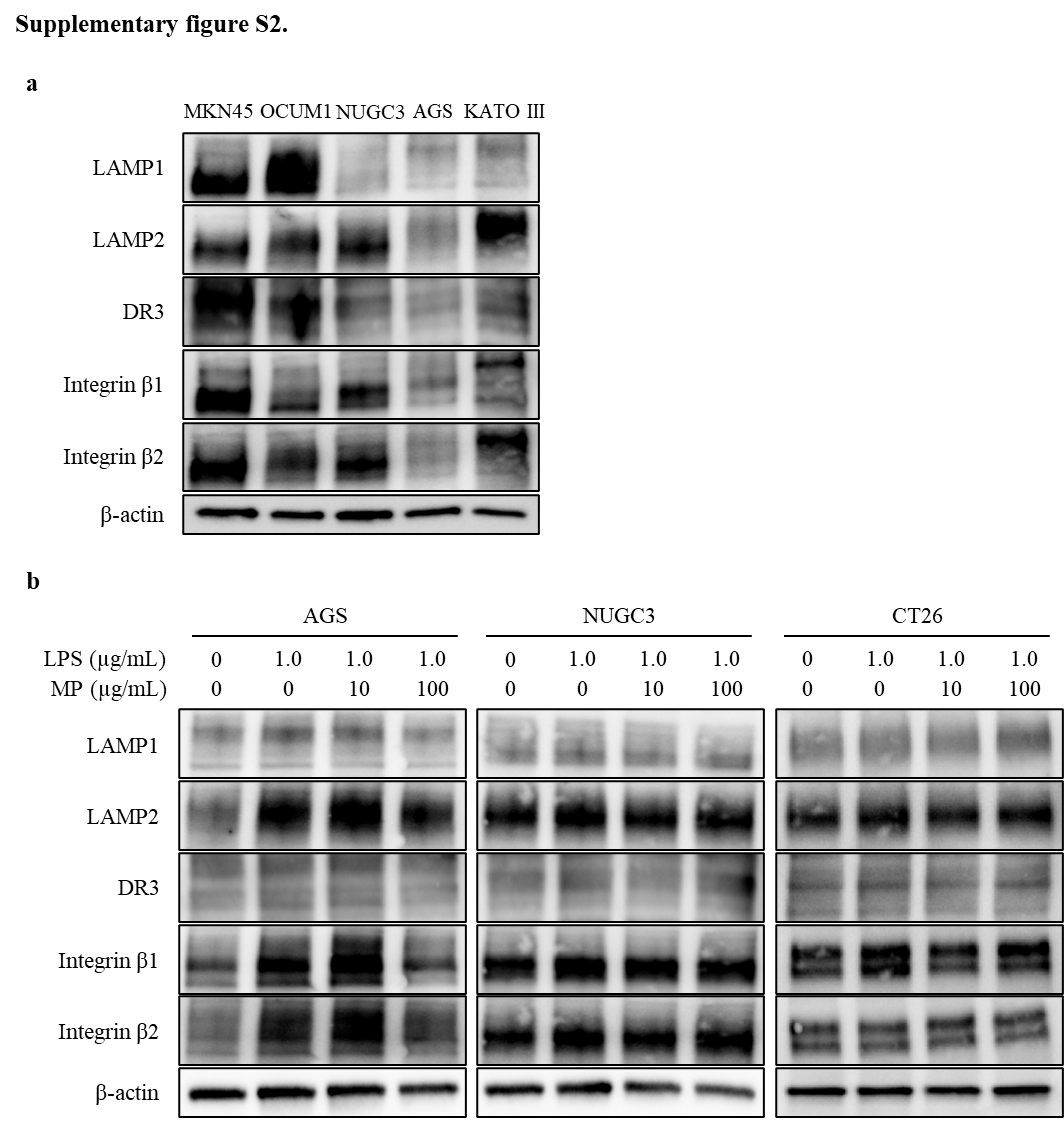
**

**
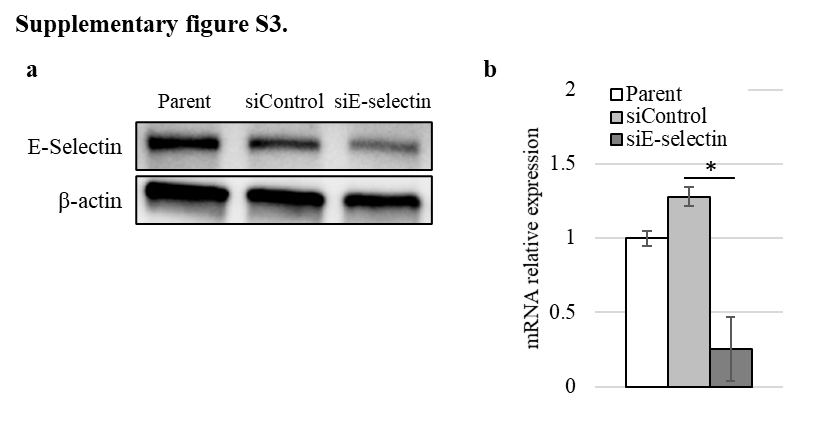
**

**
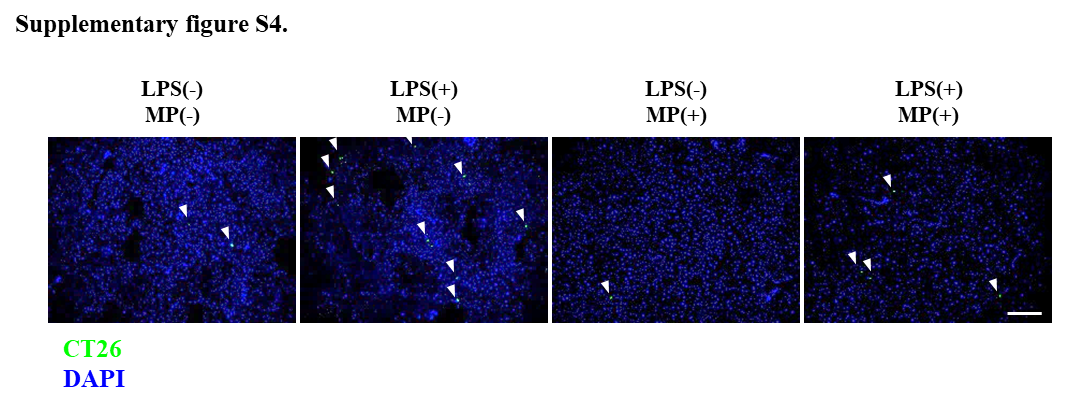
**

**
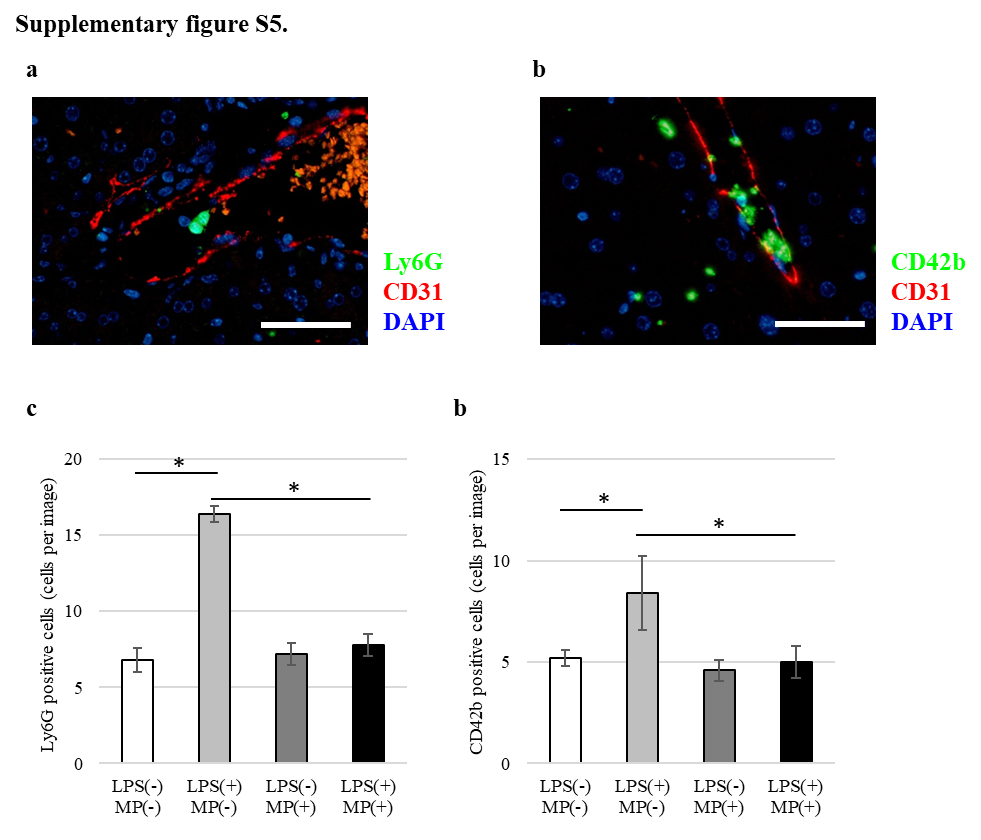
**

**Supplementary figure S6.**

**a**

**
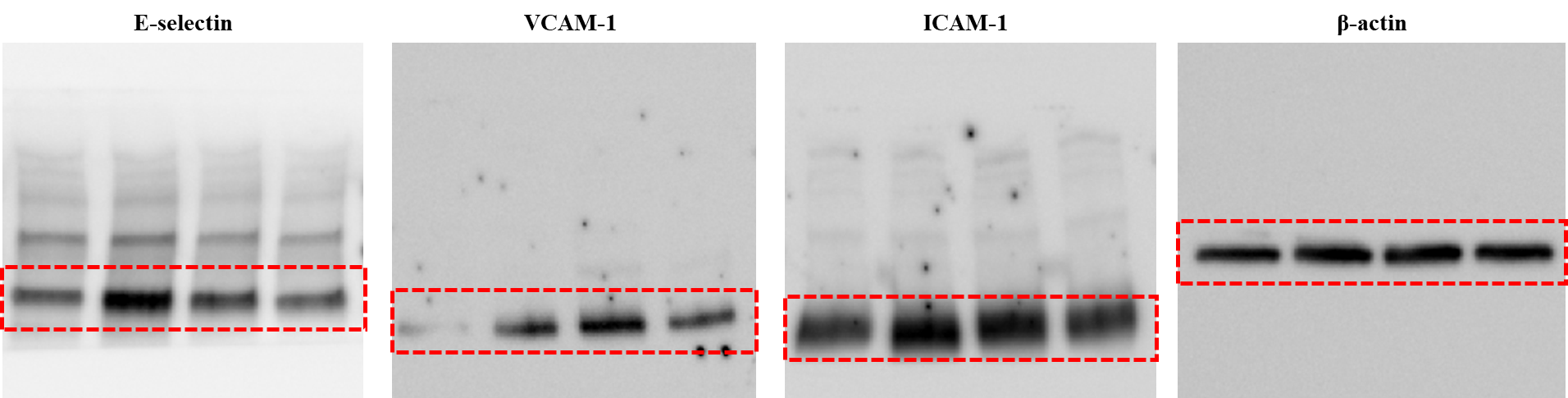
**

**b**

**
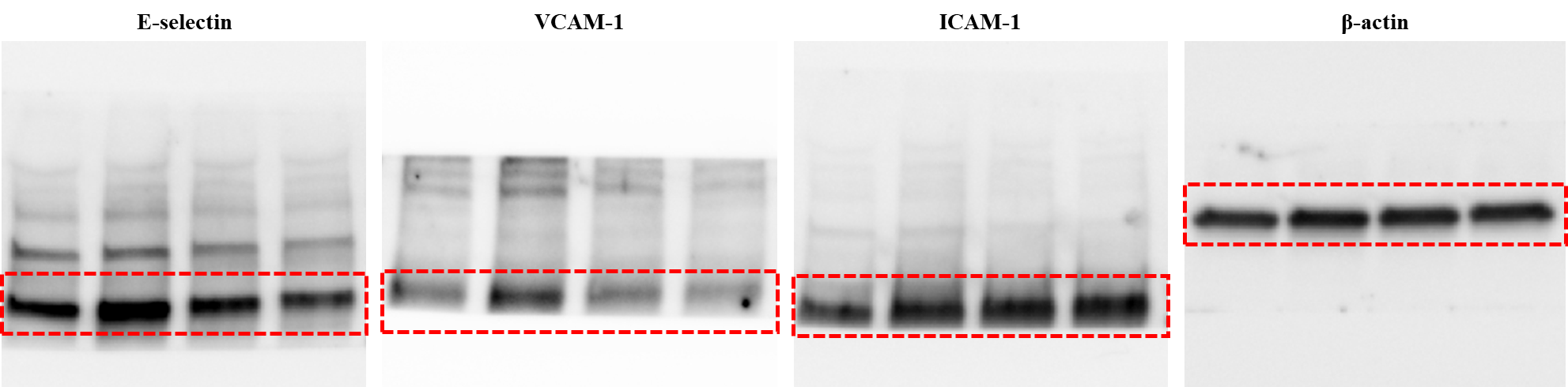
**

**c**

**
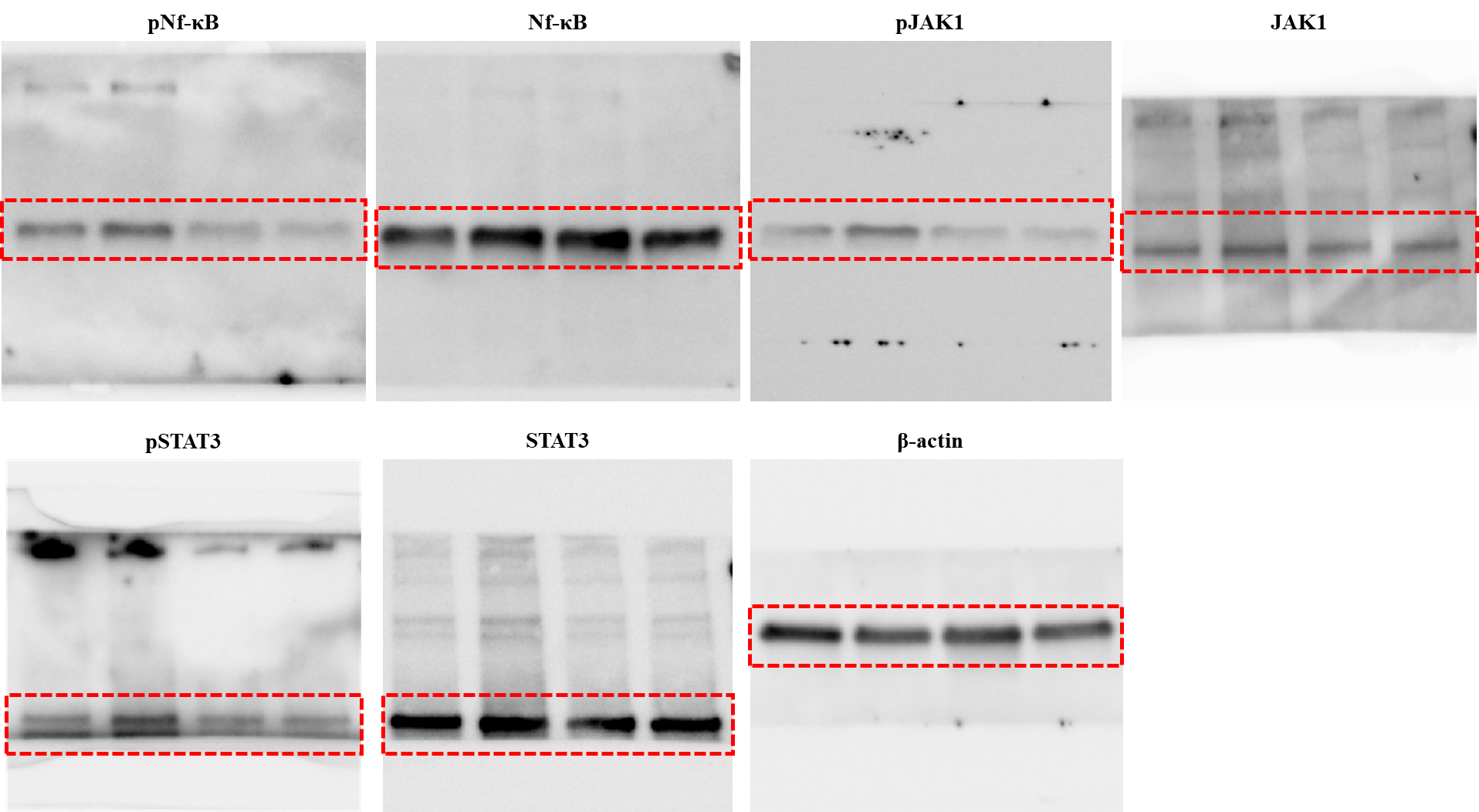
**

**d**

**
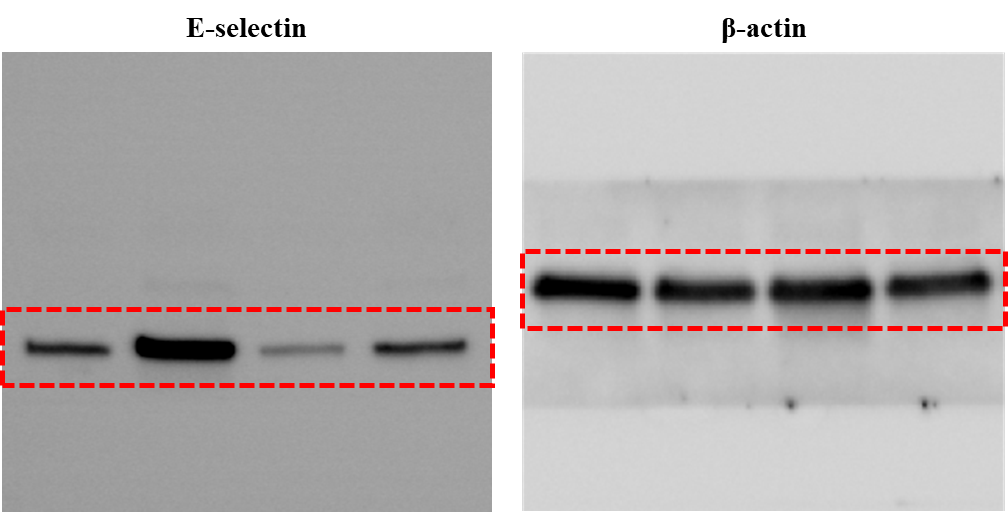
**

**e**

**
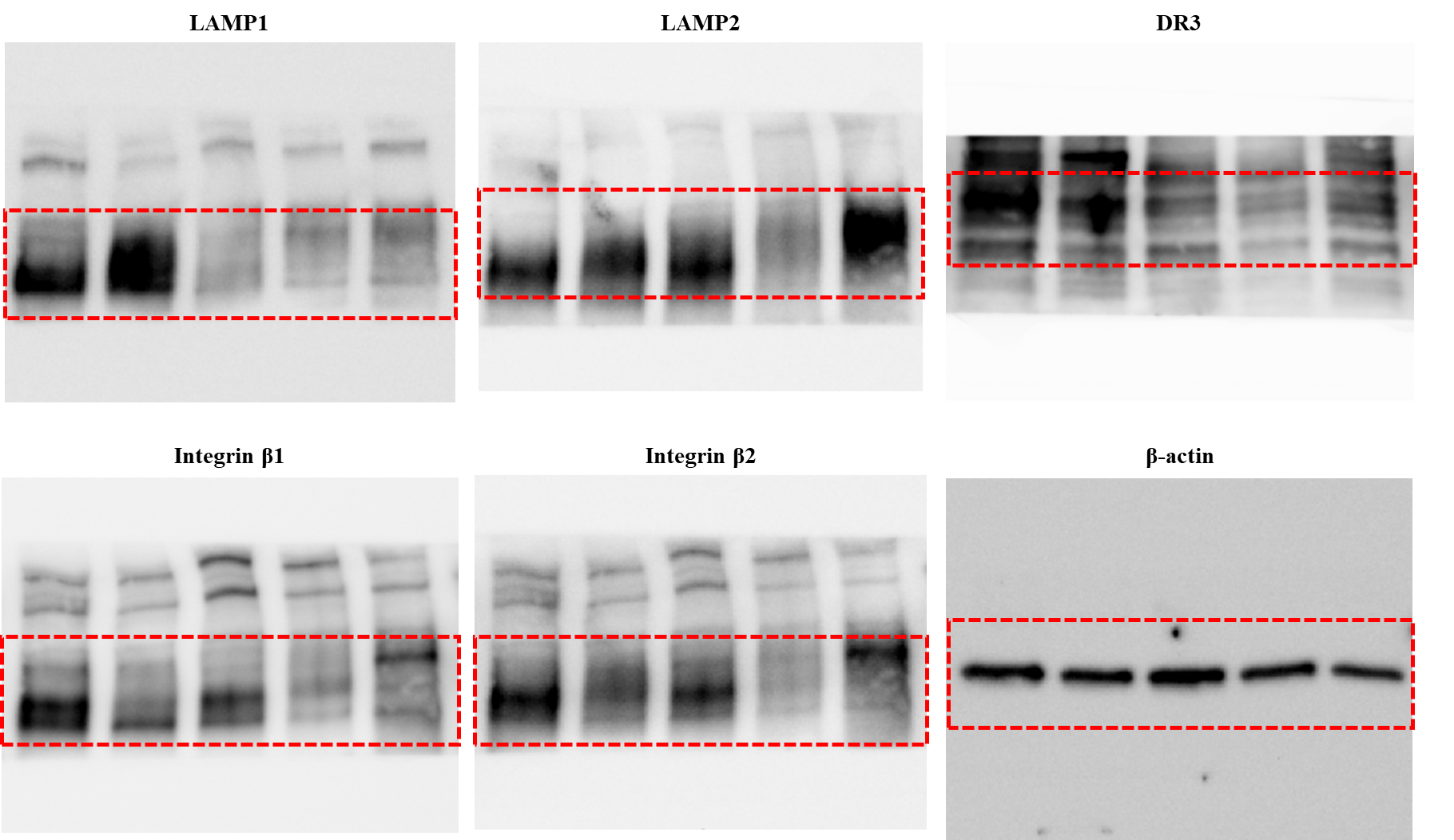
**

**f**

**
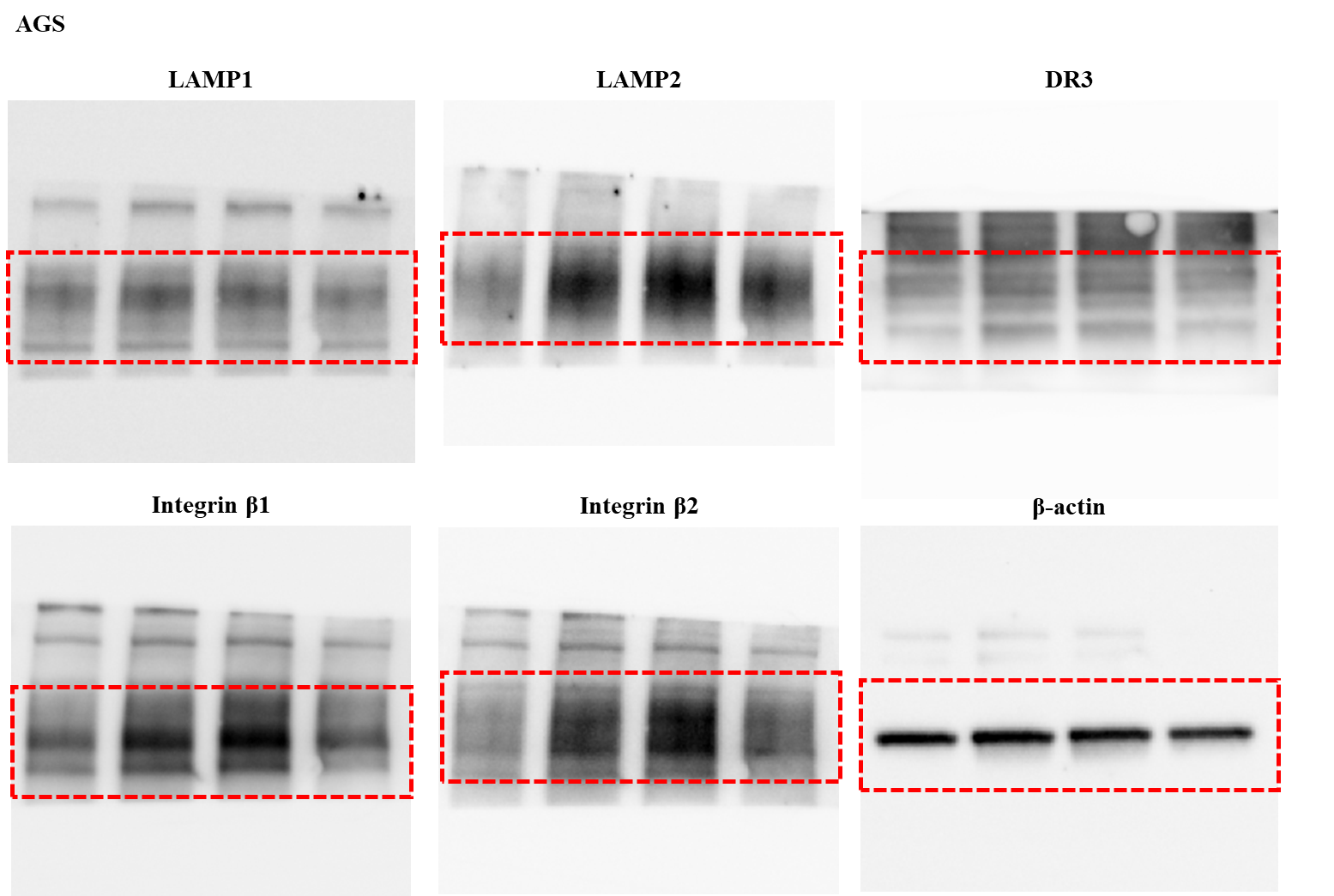
**

**
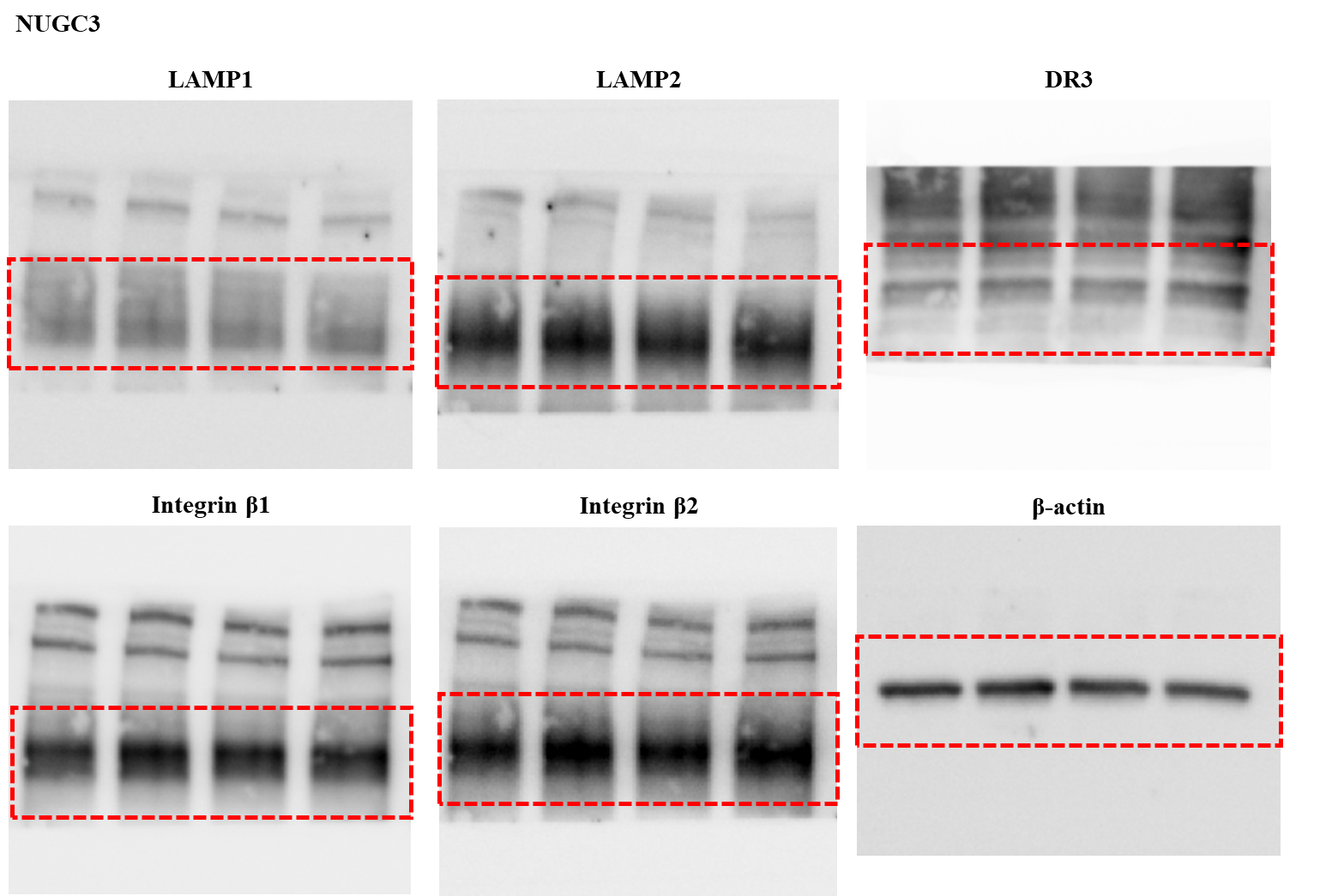
**

**
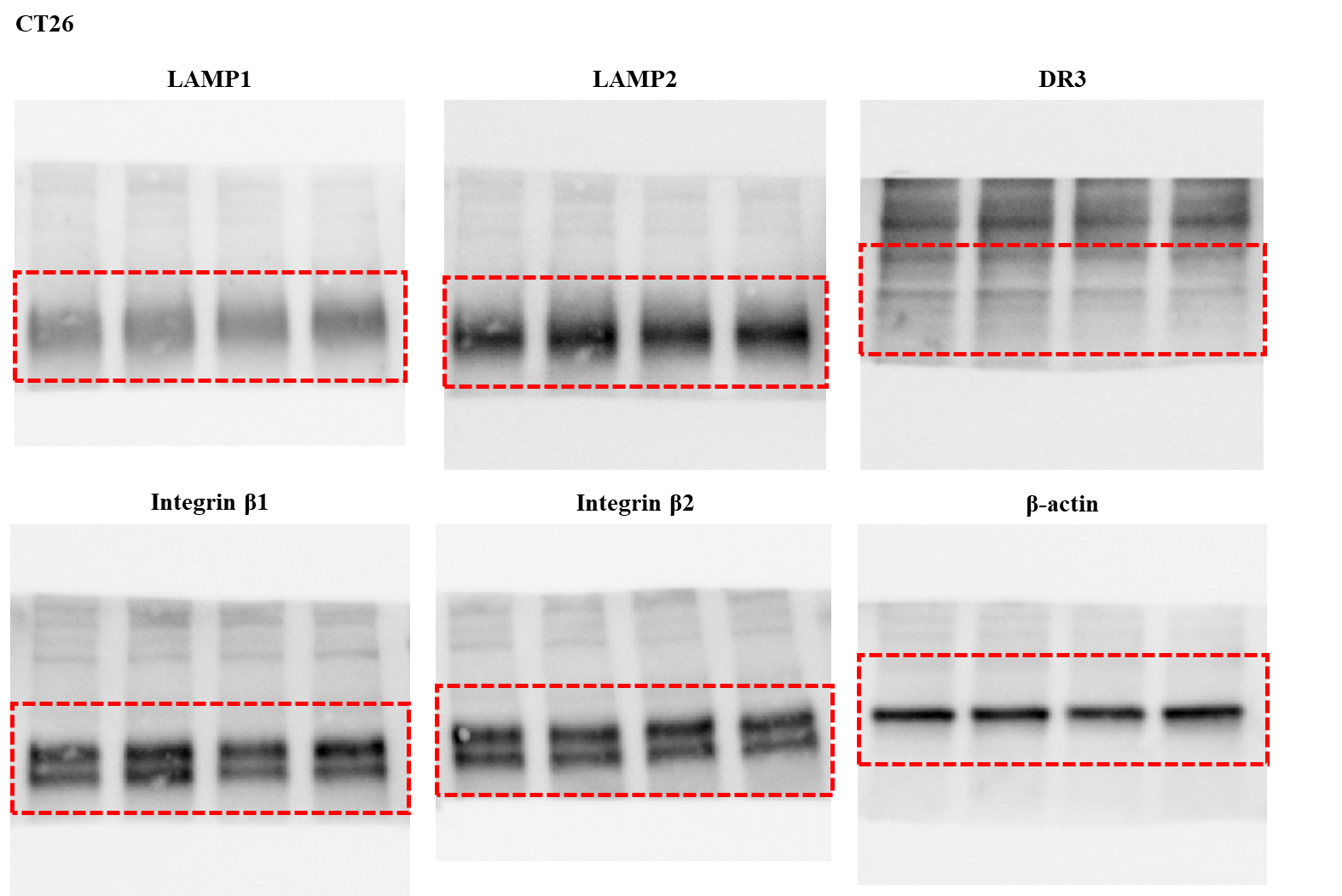
**

**g**

**
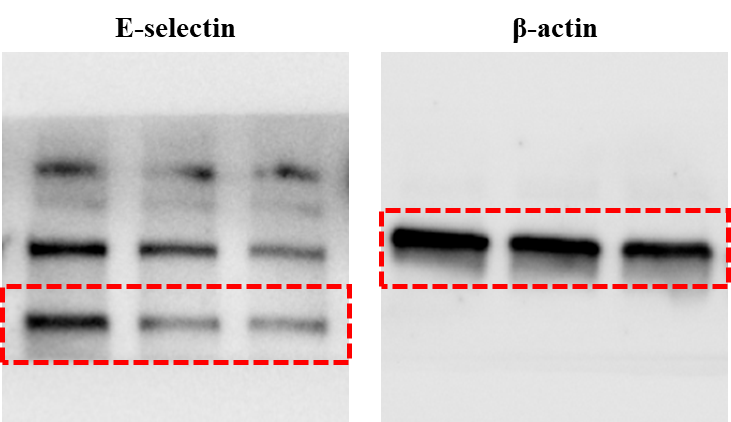
**
